# Selections and Abstracts

**Published:** 1897-06

**Authors:** 


### SELECTIONS AND ABSTRACTS.

---

### The American Pediatric Society's Report on the Collective Investigation of the Antitoxin Treatment of Laryngeal Diphtheria in Private Practice, 1896–1897.

In this second and supplementary investigation, the aim has been to ascertain: (1) What percentage of cases of laryngeal diphtheria recover without operation under antitoxin treatment? (2) What percentage of operated cases recover? The report now submitted may properly be limited to answering these two inquiries.

In order to reduce sources of error, it was desirable to bring together a large number of cases, from widely distributed localities, from many different observers and operators, and from a period of time including all seasons of the year. All returns have been examined by the committee, and only such cases accepted as bore satisfactory evidence that they were the first of all diphtheria, and secondly that the lesion had invaded the larynx.

A total of 1,704 cases of laryngeal diphtheria are ours for present study. A few cases (228) had not satisfactory evidence that there was laryngeal involvement; indeed, some were reported through misunderstanding the fact that only laryngeal cases were wanted, and a few were reported in which there was no mention that antitoxin was used. These cases are, of coure, not included in the number referred to above; 218 of them ended in recovery and only 10 were fatal.

In a total of 1,704 antitoxin-treated cases of laryngeal diphtheria, there was a mortality of 21.12 per cent. (360 deaths).

*Cases not Operated on.*—The first inquiry of the circular was: What percentage of cases of laryngeal diphtheria recover without operation under antitoxin treatment?

Of 1,704 total cases, 1,036 were not operated upon (60.79 per cent.). Of these most did not require operative interference, a few cases were thought to require it, but operation was refused. All cases are included, and it will be noted there are no eliminations.

Among the 1,036 not operated on, there was a mortality of 17.18 per cent. (178 deaths), or, to answer the inquiry of the circular exactly, of 1,036 cases not operated on, 82.82 per cent. recovered (or 858 cases).

As good as is this percentage of recovery in so large a number of cases of diphtheria of the severest type, it is believed it is not as good as it ought to be. Cases of laryngeal diphtheria not requiring operation, according to the testimony of consulting intubationists, are seldom heard from a second time, and less often find their way into reports. It was formerly estimated that about 10 per cent. of cases of laryngeal diphtheria recovered without operation. The present report shows that in 1,036 cases 82.82 per cent. recovered.

*Cases Operated upon.*—In analyzing this class of cases, it is believed a more exact conclusion as to the value of the antitoxin treatment can be arrived at than in the non-operative.

There will be entire harmony of opinion as to the severity of laryngeal diphtheria which requires operative interference. In the early days of intubation it was customary to speak of the percentage of recoveries, and 25 per cent. and 27 per cent. were considered good results. In the last report the recoveries had crept up so high in the 100 cases that it seemed more natural to speak of the percentage of mortality.

In this connection it is interesting to inquire what were the most reliable statistics of intubation, taking cases as they occurred, without selection, in pre-antitoxin days. In 5,546 intubation cases collected by McNaughton and Maddren in 1892, the mortality was 69.5 per cent., or to bring the facts into line, 30.5 per cent. recovered.

In a total of 1,704 laryngeal cases there were 668 cases operated upon. In the 668 there were 182 deaths, or a mortality of 27.24 per cent. In the former report, in 553 intubated cases the mortality was 25.9 per cent. In approximate figures there is a difference between 27¼ per cent. and 26 per cent.

*Summary.*—Sixty thousand circulars were distributed throughout the United States and Canada.

Time allowance, the eleven months ending April 1, 1897.

Whole number of cases in this report,1,704; mortality, 21.12 per cent. (360 deaths).

The cases occurred in the practice of 422 physicians in the United States and Canada.

Operations employed:

(*a*) Intubation in 637 cases; mortality, 26.05 per cent. (166 deaths).

(*b*) Tracheotomy in 20 cases; mortality, 45 per cent. (9 deaths).

(*c*) Intubation and tracheotomy in 11 cases; mortality, 63.63 per cent. (7 deaths).

Number of States represented, twenty-two, the District of Columbia, and Canada.

Non-operated cases, 1,036—60.79 per cent. of all cases; mortality, 17.18 per cent. (178 deaths).

Operated cases, 668, or 39.21 per cent. of all cases; mortality, 27.24 per cent. (182 deaths).

Two facts may be recalled in connection with this paragraph. (1) That before the use of antitoxin it was estimated that 90 per cent. of laryngeal diphtheria cases required operation, whereas now, with the use of antitoxin, 39.21 cent. require it. (2) That the percentage figures have been reversed, formerly 27 per cent. approximately representing the recoveries, whereas now, under antitoxin treatment, 27 represents the mortality. To put it in other words, before the use of antitoxin, 27 per cent. recovered; now 73 per cent. recover, and this in the severest type of diphtheria.

The present report will strike many members of the Society as revealing a mortality too large in each of the two classes. The mortality is large—larger than the personal experience in private practice of many would expect.

The reasons for this are (1) that antitoxin is still used too late, either from procrastination on the part of the physician, or objection on the part of the friends, or (2) in a half-hearted way, which shows itself in doses from one-tenth to one-fourth as large as they should be. In truth, both the physicians and the friends of the patient are timid.

This report, it must be admitted, shows too large a mortality.

In the opinion of the committee it is a larger mortality than will ever be shown again. Antitoxin is gradually being used earlier in the disease, and it will soon be used in sufficient doses.

To the Society the committee desires to say that it has sought to carry out their wishes in putting antitoxin on trial, to accept no testimony that did not bear the stamp of reliability, that it has employed the methods approved in the case of the first investigation and report, and that it has confined its work to definitely answering the main questions which the Society and profession now have in mind. Points that were settled in the first report and have since been corroborated by general medical literature are not again taken up.

If the committee is asked to put forth the three most valuable points established in this eleven months' work, they are:

1. The mortality of laryngeal diphtheria at present rests at 21.12 per cent.

2. That 60 per cent., approximately, have not required intubation.

3. That the mortality of operated cases is at present 27.24 per cent.

*The Committee Recommends:* Antitoxin should be given at the earliest possible moment in all cases of suspected diphtheria.

*Quality.*—Of the products on the market some have, by test, been found to contain one-half to one-third the antitoxin units stated on the label. Select the most concentrated strength of an absolutely reliable preparation.

*Dosage.*—All cases of larnygeal diphtheria, the patient being two years of age or over, should receive as follows:

First dose—2,000 units at the earliest possible moment.

Second dose—2,000 units, twelve to eighteen hours after the first dose if there is no improvement in symptoms.

Third dose—2,000 units twenty-four hours after the second dose, if there is still no improvement in symptoms.

Patients under two years of age should receive 1,000 to 1,500 units, the doses to be repeated as above. (W. P. Northup, Joseph O'Dwyer, L. Emmet Holt, Saml. S. Adams, *Committee.*)—*Medical News.*

### WHEN TO CALL A SURGEON IN APPENDICITIS.

The remarks which I shall present to you this evening are intended especially for the family physician, who does not operate himself, and who does not wish to assume the sole responsibility of deciding those important questions peculiar to appendicitis. It falls to the lot of the general practitioner in the great majority of instances to see the victims of this malady in its early stages, and it is scarcely too much to say that not infrequently the result is largely determined by the measures adopted at that time. . .

In as brief a manner as possible I will now consider the particular conditions and circumstances in which a surgeon should be called in consultation.

1. In cases of the fulminating variety of appendicitis, in which the symptoms are always grave, and generally becoming worse from hour to hour to an alarming degree, it is scarcely possible to obtain surgical aid too early in the disease. An operation may, or may not, be necessary, according to the condition of the patient; but, from the very nature of things, an experienced surgeon is the best able to decide that point. No one would think of operating upon a person while in a state of profound collapse. The proper treatment for one in that condition is hypodermic and rectal stimulation and external heat. If he cannot be rallied in that way, he certainly cannot be by an operation. Profound prostration, which differs from collapse in that the clammy sweats, cold extremities, flickering pulse and sighing respiration are lacking, may call imperatively for an immediate operation. By speedily relieving the system of the pent-up septic materials the vital powers are thus enabled to rally and to regain their normal condition. Hence in these cases of sudden and severe seizure, you want all the aid you can get, and you want it at once. Don't wait till to-morrow.

It may be said here, that, contrary to what might be inferred from some writers of the present day, an operation, however early it may be performed, will not save every case of appendicitis. A small proportion of the victims of this disease will perish whatever may be the treatment, and however promptly and thoroughly it may have been adopted. Operations performed within twelve hours of the onset of appendicitis have failed to give the slightest relief. And yet this fact should not discourage early operations, when they are indicated, for in very many instances the convalescence dates from the time of the operation. It is simply a reminder that too much must not be expected from surgical measures.

2. The greater proportion of cases of appendicitis met with in ordinary practice will probably come under this head. The symptoms are moderate in severity at first, but steadily grow worse every twelve hours or so, with perhaps occasional remissions. These cases are often very deceptive. The patient does not seem to be very sick. With an occasional opiate, or perhaps without anything of the sort, he suffers little pain; and the attendants and friends are loath to believe that a serious, if not a dangerous, process is going on in the abdomen, until the vital powers are so exhausted that the patient's chances for recovery are very materially diminished.

In these cases I should strongly urge that the surgeon be called not later than the third day. Very likely the symptoms will be only of moderate severity at that time; but the consultant can form a much more correct and satisfactory idea of the character of the disease before its manifestations have reached the point of danger. He will also be better prepared to attack the case at the proper moment; in fact, he can choose his own time for interference. It is hardly possible to attach too much importance to this point. If the symptoms do not show marked amelioration by the third day, further counsel should be sought, not that an operation may be demanded at once, but that due preparations may be made, and that no time may be lost when time is important.

3. A patient has a moderate attack of appendicitis. He is to all appearances improving, when, without any definite reason, the symptoms are aggravated. He has a relapse, and has to make another start in his convalescence, only to experience the same chain of events, sooner or later, to prevent his recovery. The patient, and oftentimes the physician, is inclined to attribute these relapses to over-exertion, an error in diet, catching cold, etc. While these factors may occasionally act as exciting causes, yet the essential feature to be borne in mind is a damaged appendix.

Every exacerbation mean an extension of the morbid process, and leaves the patient a little weaker and a little worse off than he was before. The time may come when he fails to rally, and he then becomes an invalid from chronic appendicitis.

These cases are not quite free from danger, and sound judgment is required to decide the proper period for interference. Each case must be managed according to its condition and peculiarities, yet it may be said, as a rule, that two, or, at most, three or four, relapses, according to the severity, are sufficient to call for removal of the appendix. I presume that every surgeon feels that he has delayed action too long in occasional instances, but I have yet to meet one who has cause to regret a premature operation under these circumstances. It is better to err on the side of safety.

4. No variety of appendicitis is more embarrassing to the attending physician or to the consultant than the following: A man is seized with a sharp attack of pain in the umbilical region, accompanied by tenderness. It knocks him out, so to speak, doubles him up, and sends him to bed. The symptoms may be severe enough to produce a moderate collapse. An opiate under the skin gives him complete relief for several hours. Seen for the first time during this period, the patient seems to have little or nothing the matter with him. No pain or tenderness, no disturbance of pulse or temperature. Everything is quiet under the sway of the "divine poppy." In many instances this lull in the symptoms is deceptive, as in from twelve to twenty-four hours they return with increased violence. This is usually the signal for calling the surgeon, and it goes without saying that he should be prepared to operate at once if necessary.

5. And, finally, we have the recurring cases of appendicitis to consider, which differ from the relapsing variety in the fact that patients are apparently well between the attacks. Recently a gentleman consulted me for the following symptoms: Five or six times during the past year, after a hearty meal he has been suddenly seized with a severe pain in the umbilical region, which gradually worked down into the vicinity of the appendix. The pain is accompanied by marked local tenderness and inability to stand erect. It lasts about twelve hours, but the tenderness persists for three or four days, or even longer. He is weak and exhausted for several days—much more so than would be expected after an ordinary attack of colic or indigestion. This man is strong, robust, and is entirely well between these attacks. In my opinion, he is suffering from a mild form of recurring catarrhal appendicitis. Now comes the important question of treatment. Is an operation necessary, and if so, when shall it be performed? The temperament of the surgeon and of the patient is a factor in deciding these questions. If the attacks are increasing in frequency or in severity, there can be little doubt as to the necessity of an operation; and the sooner it is done the safer and better for the patient. On the contrary, if the intervals are increasing in length, and the symptoms becoming less severe and of shorter duration with each attack, it might be well to wait awhile before resorting to a radical cure, as a certain number of these cases do eventually wear themselves out, so to speak, and entirely disappear, thus coming under the head of "appendicitis obliterans." In the opinion of the writer, six attacks of appendicitis in one year, even if mild, are quite sufficient to justify an operation for the removal of the exciting cause.

Not a few physicians entertain the idea that there is no occasion for calling the surgeon until a tumor has formed, indicating that the inflammatory process is limited in extent and has ceased spreading. That this idea is erroneous and misleading is abundantly proved by the fact that in very many cases no tumor is ever found, and yet the convalescence dates from the moment of operation. It is often impossible to detect a tumor, even when present, by reason of its depth in the pelvis, its shape and size, as well as the thickness and rigidity of the abdominal walls. The presence of a tumor does not of itself indicate an operation, nor does its absence preclude it. In fact, the indications for treatment are influenced very little by this symptom. A tumor shows the variety of the inflammatory processes going on in the peritoneal cavity, and also indicates the site of the incision. The plan of treatment is based on other and more important factors in the condition of the patient. If he is growing steadily worse, radical means are called for, and the more rapid is the progress of the symptoms, the earlier are effective measures demanded.

In closing, I can but repeat what has already been said, that from a surgical standpoint it seems wise and prudent that every case of appendicitis of any severity or duration should be seen in the early stages by one accustomed to operating for this affection, as well as to deciding the many difficult questions which are constantly arising throughout its course.

If the initiatory symptoms were severe; if they are steadily growing worse; if they relapse, or come to a standstill; if the patient is sick, weak, irritable, impatient, restless; and especially if he cannot pass wind, or is inclined to nausea, vomiting, hiccough, or delirium—then I urge you to be vigilant and prompt in calling for surgical aid, for reasons already mentioned. You will never be criticised for calling it too early or too often. The serious character of the affection, the sudden onset, the insidious course, the rapid and unexpected variations, the startling collapses and excruciating pain liable to occur in this affection, as in almost no other, will protect you from these charges. And should the result be unfortunate, you and the friends will have the lasting satisfaction of knowing that you have done your whole duty in the matter, and are in no way responsible for the disastrous termination.—DR. G. W. GAY, *Boston Med. and Surg. Journal.*

---

### REPORT ON MALARIAL HEMATURIA.

This disease is one of particular interest to physicians of the Southern Coast and Gulf States, and they will welcome any additional light which may be thrown upon it.

More than a year ago a committee was appointed by the Tri-State Medical Association of Mississippi, Arkansas and Tennessee, to make a collective investigation of malarial hematuria. This committee went to work apparently in earnest, sending out five hundred forms of case reports to the same number of physicians, requesting reports of cases in detail. From the report of this committee (*Memphis Medical Monthly*) we learn that the doctors appealed to were surprisingly indifferent to the objects of this investigation. However, reports were made upon fifty-seven cases, and from an analysis of these the committee finds:

First, that malarial hematuria, so-called, is a disease of the prime of life, the ages ranging from three to fifty years, and twenty-four years is the average age. No occupation seems exempt; for the patients come from every class found in the parts of the country from which reports were received, and in due proportion. The colored race is not entirely exempt; for there were fifty-two white and five colored cases reported.

Forty-one of the cases were males and sixteen were females, the only conceivable reason for the great predominance ot males being better treatment accepted by the females. The male is more often exposed to the early morning air, it is true; but few of us can note that the women are not as frequently attacked with acute malarial manifestations as are the men.

All of these fifty-seven cases occurred between July and March, inclusive; July and March each giving one case, August 12, September 10, October 17, November 5, December 4, January 3, and February 4. So August, September and October are the principal months to expect resulting hematuria, though earlier months really do the damage.

The drinking water seems to cut no figure. Thirty-five reported wells, presumably driven wells, as the source of the water supply; three cisterns, six Mississippi river water, and thirteen not given. And this must be about in proportion to the total use of such sources of drinking water.

Fifty-one cases were reported as being subject to malarial attacks for a greater or lesser length of time. Six cases were reported as not being so subject. It is almost beyond experience to hear even one case occur without previous malarial manifestation.

Of the fifty-one cases reported as having a history of malarial poisoning, in every case it was the intermittent form that occurred. No case is reported of hematuria developing during a remittent or continued fever. There is food for thought in this view of the present division of opinion among the bacteriologists as to the identity of the different forms of the germ of malaria.

Of the fifty-seven cases the onset of lysemia was marked in thirty-nine by a chill, in nine by fever only, and in nine no data. Nausea and vomiting present in all but three cases. The urine was reported a pokeberry-juice color in twenty cases, black in twenty-five, wine color in seven, chocolate color in three, and poke juice changing to chocolate in two. In all except two cases the quantity decreased with each passage of urine until symptoms were relieved. In these exceptional cases, two in number, the urine passed was of such great quantity and so largely blood products as to practically cause exsanguination. Jaundice occurred perceptibly in fifty-four cases; not present in three.

In thirty-six cases quinine had been positively given before the attack; in only one case was there certainly no cinchonism at the time of the attack, the remaining cases giving no data. The treatment is more in line than one would expect. Forty-two cases were treated on the eliminative plan without quinine, and thirty-eight recovered and four died; the four fatal cases were from what may be called complications; at least three of them were in such general health that they would have succumbed to almost any acute disease. In thirteen cases quinine was the basis of the treatment, and eight recovered and five died.

We have of recent cases but five treated with quinine as a main reliance; of the five four died, mortality eighty per cent., the rate given in nearly all old text-books; while the forty-two cases treated with calomel, with hyposulphite of soda, and with salts and turpentine only gave four deaths, a mortality rate of nine and three-fourths per cent.; total recoveries forty-six, deaths eleven, mortality nineteen and one-third per cent., by all treatments.

As regards the methods of treatment four physicians advocated the routine use of quinine; against the use of quinine sixteen; of whom three admitted that there were certain very rare cases complicated with malarial fever in which quinine might be properly but guardedly used. Three of the physicians regarded hyposulphite of soda as specifically antimalarial; the rest did not so agree, though most admitted its value as a purgative and eliminative.

It would seem, from forty-two cases being treated without quinine to five recent cases with quinine (only two of which in past year),

4

that ninety per cent. of the profession are able to see that the so-called disease hematuria is not a malarial paroxysm, but a pathological condition resulting from many malarial paroxysms in which as a rule the malarial poison has spent itself and quinine is not needed, but elimination and rebuilding are the indications. From the mortality from this plan, of nine and three-fourths per cent. mostly accounted for by complications, and the mortality where quinine was used in recent or noted cases of eighty per cent., it would seem plausible that the said ninety per cent. of the profession were correct in their conclusions.

Leaving the cases, we have, of the observers giving opinions, sixteen to four against quinine—eighty per cent. of the physicians. But a few of these recognize the fact that a malaria may complicate lysemia, as well as it may complicate typhoid or a broken leg. These admit that in such cases, which they declare to be rare indeed, quinine may have to be used. Almost to a man, they also declare the unfortunateness of such a complication; for they say that in most cases quinine will do harm. Not only does it require elimination by the kidneys and cause a tendency to suppression of urine, but it has been proved that quinine in toxic doses causes diastatic arrest of the heart's action. This is of moment; for in no disease is the heart's action to be more guarded, and even ordinary antiperiodic doses may do harm to a failing heart. Bonard, Arvedi, Magendie, Monneret, Melier, and Baldwin declare that in animals killed with quinine the blood is found to be dark, defibrinated, fluid, and incapable of forming a clot. Briquet denies this, but found that a continued use of the drug augments the proportion of fibrin, but lowers that of the red blood corpuscles—the very site of the pathology of this condition. So with the kidneys, the heart and the integrity of the red blood corpuscles theoretically threatened, and the actual occurrence of fresh disintegration of blood cells and suppression of urine determined many times empirically, as caused by quinine administration—the physician with a case of lysemia complicated with malarial paroxysms, and therefore needing quinine exceedingly, has no time for sleep. He must either give quinine and increase the amount of lysemic action and thereby in some unknown way cause a subsidence of the malarial complication too permanent to be due to quinine alone, and then hasten to eliminate and protect his patient from the danger of suppression, and if this does not occur give quinine at intervals until a tolerance be established, or he must seek a substitute, and that is hard to find.

Let us look back once more to the figures. Fifty-one cases gave a previous history of intermittent. Now, there is a glorious chance for prophylaxis. You can blow out the match, when the whole fire department may not be able to put out the house on fire. Why have any hematuria? Why not cure all these intermittents in time? Some of them you never see; but if you are called to see a case of lysemia in a family where your word is respected, the person of a patient whom you have treated for intermittent, and that patient being a reasonable being, just half way carrying out your instructions, you ought to be ashamed to look that patient in the face. With such an absolute specific for intermittent as quinine, and with half the obedience usually accorded the family physician, there is absolutely no excuse for the occurrence of a case of hematuria in your regular practice. Outside cases and unknowns and chill-tonic fiends may have it and send for you, and you can go with a clear conscience to those cases; but in those cases who have looked to you for care daily, you are as much to blame as if they had smallpox through your failure to insist on vaccination.

Give quinine properly in antiperiodic doses laid down in the common text-books, look up the Italian and German dosage if you feel lonesome, and cease to humor the patient with the little, time-killing doses becoming so customary. There is one word for the dose of quinine, and that is, *enough;* not enough to cause tinnitus, not enough to cause slight deafness only, though that may be and is in most cases sufficient, but *enough to keep off the paroxysm* Remember you are not merely dosing the patient; you are poisoning malarial germs. Give enough for that; it will not require a dose lethal to the patient.

### THE TREATMENT OF CANCERS.

At the late meeting of the Association in Macon, two interesting papers on this subject were read, one by Dr. Slack, on the "Use of Pyoktanin in Malignant Growths," the other by Dr. Morgan upon the "Treatment of Cutaneous Cancers." (See page 178, May issue.) Part of Discussion, by Dr. M. B. Hutchins, Atlanta:

My experience with the use of pyoktanin has been been confined to one case, which I treated about six years ago. The case was one of multiple epithelioma of the face and of the external auditory meatus, where I had removed a large ulcerating epithelioma from between the eyes at the root of the nose, which did not heal, and at that time I began to notice some reports regarding the use of blue and yellow pyoktanin. I used the blue pyoktanin on the unhealed operated wound, which was cancerous. The only effect I saw from its use was a slight restraint upon the virulence of the growth, and there was an increased effort on the part of the wound to heal. I continued the treatment for three or four weeks, and had to wash my hands in vinegar every time I got the pyoktanin on them. Finally the patient died of cancer of the lung, and his face was so badly stained that the undertaker was curious to learn the cause. From this time on I did not see enough favorable reports in literature to follow up the plan of treatment, besides not obtaining a satisfactory result in the case I treated.

Dr. Slack should be congratulated on what he has done in the palliative treatment of these cases, because a great many of them, perhaps the majority, are cancerous and are inoperable, and we have got to treat them as we would any other septic diseased surface. By strict antisepsis we are enabled to control in a measure the destruction which is going on from the poisons generated, and in this way we can control the rapid breaking down of the disease.

As regards the injection of pyoktanin around the carcinomatous growth and to prevent cancerous cells from entering the circulation, it is possible for offshoots of this disease to be in the lymphatics, entirely away from the apparent margin of the cancer, and it is possible by injecting the pyoktanin around the periphery of the tumor or beneath it, to push some of the cancer cells into the circulation, thus transporting the cells to other parts of the body. So the objection where we make the injection directly into or through the tumor still holds, or where there is any possibility of the cancer cells having commenced to pass through the lymphatic channels.

I think Dr. Morgan has obtained some very excellent results with his caustic applications, and personally I wish to thank him for the paper he has given us regarding the pathology and treatment. I believe Dr. A. R. Robinson, of New York, was the first physician in this country to work out the subject of pastes and caustics and their effects upon cancerous disease of the skin. He has written a great deal upon this subject, and claims to have secured such results, as Marsden did before him, with the paste and caustics, as could not be obtained with the knife. At one time I was tempted to become a complete convert to Robinson's method of treatment, but from increased experience I have come to believe that it will not do to consider that all of these cases of superficial epithelioma must be treated by strong caustics, or any special kind of strong caustic, which is a very painful method of treatment, in order to destroy the growths.

There is one feature in connection with the treatment of epithelioma which is of paramount importance, and that is to remove all of the diseased tissue. If the physicians who have had failures with the knife in the treatment of these cases had continued to use the knife and followed it out more thoroughly by going just a little farther beyond the diseased area, then filling the wound either by skin grafts, skin sliding, or a plastic operation, I believe they would get as good effects from the use of the knife as from caustics, in many cases.

As regards the action of the inflammation in destroying the cancer cells, as is claimed by some—and, understand, I use caustics more than I do the knife in these cases—if the inflammation does destroy and cut off the little infiltrating cells and kills them, why have the experiments of Coley with the erysipelatous streptococci and the bacillus prodigiosus so utterly failed? They must produce some inflammation. In ordinary cases of epithelioma of the skin I prefer largely the use (under proper cocaine anesthesia) of the solid stick of caustic potash, and have obtained good results with it. I have also obtained good results with the Paquelin cautery, as well as with the use of Marsden's paste, and yet I have seen every one of the methods fail, bringing us back to the point that I mentioned a short time ago, of not getting, or removing all of the disease. It seems to me that the question of inflammation does not explain the whole thing. I think it is just the important point of going far enough with the caustic, cautery, or any of the applications which are used. Caustics are not wholly under your control, and you get a certain amount of destruction that you do not aim for.

One serious objection to Marsden's paste is the length of time it must be left on the surface, and the pain it produces. You may, of course, have cocaine in your paste, but after a while the cocaine will lose its effect, and the patient then suffers. In the case of an old woman, I gave her a hypodermic injection of morphine, one-half grain, and to all intents and purposes she was dead for the next twelve hours, and I could continue the use of Marsden's paste on her nose for that length of time without causing pain. I do not wish to be understood as condemning pastes and plasters altogether, but I do not think we should consider them the only method of treatment in these cases. If we have cells which have become separated from the original epithelioma and have passed into the lymph spaces, and the spaces behind are practically closed up, the caustic does not reach these cells, which seep along through the skin. In such cases we get a recurrence of the growth outside of the margin of the original treatment. In cancer of the breast, where we have involvement of the glands, we cannot expect to reach by caustics those cancer cells which have passed on to the lymphatic channels.

---

### SOME IMPORTANT FACTS ABOUT CHLOROFORM.

An interesting paper with this title was read by Dr. H. A. Hare before the College of Physicians of Philadelphia, January 6th, a portion of which follows (*Therapeutic Gazette*, February 15th):

As with other discussions in medicine, the truth of the questions as to whether chloroform causes death by respiratory failure or cardiac failure lies, as it were, half-way between the two antagonistic forces; and, further than this, the somewhat startling statement may be made that it is not directly due, in the majority of cases, to either of these causes. On the contrary, *the cause of death from chloroform is usually vaso-motor depression,* whereby the arterioles allow the blood to pass too freely into the great blood-vessel areas which are found in the capillaries and veins, and as a result the man is suddenly bled into his own vessels as effectually as if into a bowl. When it is remembered that the capillary network of the body will, with the relaxed veins, hold many times the normal quantity of blood, and when it is remembered that we can inject salt solutions into the vessels to the extent of several times the normal quantity of blood without raising the blood pressure, it at once becomes evident that the complete vascular relaxation caused by chloroform results in failure of all the vital functions, not because the drug has paralyzed the heart or respiratory center, but because these parts are deprived of blood by its stagnation in the widely dilated capillaries and abdominal veins. Recent studies by Leonard Hill on "The Physiology and Pathology of the Cerebral Circulation" showed that this was the case, for he asserts that when the blood was no longer flowing to the respiratory centers the heart was still beating, because its coronary arteries being lower down, were more easily supplied by the small blood-stream received by the heart from the veins. These studies are proved by the experiments of myself and my assistant, Dr. Thornton (*Therapeutic Gazette,* October, 1893), by every tracing of the Hyderabad Commissions, and all other tracings we have ever seen. We may conclude, therefore, that while chloroform without doubt acts as a powerful depressant poison to the respiratory center and the heart, in the same manner as it paralyzes all living protoplasm when applied in excess, that when properly given by inhalation it produces a death equivalent to that resulting from hemorrhage, which is a failure of the respiration not so much from a direct depression of the respiratory center as from its starvation of blood; and while the tendency of the drug is to depress and dilate the heart, just as it dilates the vessels of which the heart is merely a high specialized part, the failure in the pulse rests upon vaso-motor palsy, the patient becoming pulseless because the heart has not any blood to pump.

Let us see what evidence supports this view: First, we have the laboratory tracings of many independent investigators extending over many years and made in all parts of the world, all of which show a fall of blood-pressure. Among these may be named Bowditch and Minot, of Boston; Coats, H. C. Wood, Gaskell and Shore, the Hyderabad Chloroform Commissions the studies of Wood and myself in 1889 and 1890, and of myself and Thornton in 1892 and 1893. They are confirmed by Hill, who has seen the abdominal vessels engorged with blood under chloroform, the medulla almost bloodless and the heart still pumping though respiration had ceased. They are confirmed by my own experiments, in which I proved that even after the respiration had stopped and the carotid was empty, and the dog apparently dead, he could be resuscitated by visceral compression and artificial respiration, and by inversion whereby the blood left the dilated abdominal veins for the heart and brain. Again, if a needle was inserted through the chest-wall the heart was found to be beating, for the needle moved to and fro; and finally if the chest was opened the heart could still be found beating feebly—dilated, it is true, but beating.

So much for the laboratory evidence. What have we in clinical evidence? Equally positive proofs of vaso-motor palsy, and none of death being purely cardiac or respiratory. For years Chisholm, of Baltimore, and later Howard Kelley, and a large number of others have used inversion with compression of the floating ribs in artificial respiration, which has forced the blood into the chest and saved life again and again. For years the literature of medicine has teemed with reports of death from chloroform while the patient was sitting up or half recumbent, because the blood-paths being dilated this posture favored anemia of the vital centers. Again, it has been proved both experimentally and clinically that the best vaso-motor stimulant—belladonna or atropine—given before the chloroform, increases its safety, and that compression of the limbs by bandages does likewise. Finally, Hill has shown that abdominal compression also produces resuscitation by sending the blood to the heart. On the contrary, saline transfusion, which at first glance would seem to be indicated, is useless, because the dilated blood-paths will receive all the saline for a long time before they will overflow towards the heart, for as fast as the fluid flows in they dilate.

My conclusions therefore are, that while chloroform in its general depressing power depresses all vital functions, it is the question of blood-pressure which is most important, and therefore in the use of chloroform we should always keep the head low, precede the use of chloroform by atropine hypodermically, bandage the limbs if the case is feeble or already bloodless, and if necessary place compresses on the belly and apply them deeply by pressure if a failing circulation is developed.

The primary action of the chloroform is to depress the blood-pressure chiefly by its vaso-motor effect, secondly by its cardiac effect, and finally that while the drug does exercise a depressant effect on the respiratory center, the failure of this center is chiefly due to anemia. As, however, an intact respiratory center means regular breathing, we watch this function to determine the dose of chloroform actually inhaled, and because any variation in this function, as shown in irregular breathing, means that the chloroform is disordering arterial tension. Death from chloroform, then, is usually a vaso-motor death, for an intact arterial system is as important to vital function as an intact cardiac apparatus.

---

### NOTES ON THE TREATMENT OF ANEMIAS.

The evolution of organic iron compounds has been interesting both from pharmaceutical and clinical standpoints. The scale salts were the first to attract attention, and their superiority over the inorganic preparations soon became manifest. The hydrated succinate then came into favor, and later the "albuminates" were introduced—indefinite mixtures whose solutions readily coagulated, even spontaneously. In Gude's peptonate of iron and manganese we have a stable and palatable preparation, uniform in composition and therapeutic in effect. Theoretically it would seem that iron could only be of service in chlorosis; clinically this rule does not hold good. I have used pepto-mangan Gude some five years with good results, and beg leave to report the following cases:

Case 1. Mrs. C., aged 31; hemorrhage following an abortion at fifth month. Saw the patient several hours after the accident, almost exsanguinated and delirious. Hemorrhage was stopped by curetting. She rallied nicely; no transfusion. Next morning found her very weak; no blood examination permitted. Was put upon pepto-mangan Gude, tablespoonful three times a day, milk and beef tea. At the end of a month the blood count was 4,800,000; hemoglobin, seventy per cent. Although she felt entirely well, pepto-mangan was continued. At end of third month she menstruated as usual.

Case 2. Mrs. K., aged 29; had had a miscarriage two months before, but had lost very little blood. Dr. W. W. Taylor had assisted at the management of the case, and no curettement had been deemed necessary. There had been no fever, but patient was steadily losing ground. Blood count 3,200,000; hemoglobin not estimated. She received the same nourishing diet as before and pepto-mangan Gude. At end of five weeks (June 3, 1892,) her blood contained 4,300,000 red cells to the cubic millimeter (had no hemometer at the time). She was able to be up, had a fair appetite, and was gaining daily. She went North on a trip, and no subsequent count could be made, but looked better than for a year past on her return.

Case 3. Mrs. L., aged 50; "nervous break down"; had been treated unsuccessfully for two years; had also had morphine habit. Red disks, 2,800,000; hemoglobin forty per cent. Treatment: Pepto-mangan Gude (tablespoonful three times a day), strychnine hypodermically, phosphide zinc (one-fourth grain t. i. d.). After seven weeks she was able to take short walks, and had gained eighteen pounds. When she returned home the hemoglobin percentage was sixty-five; count could not be made, as I had not brought my cytometer. She was instructed to continue the treatment at home.

Case 4. Aged 35, male. Was treated at my sanitarium for opium habit, but owing to business pressure had to leave for home before fully recovered. He returned in three weeks to be "built up." Had hot and cold flashes, insomnia, was very nervous. Red cells 2,320,000, some microcytes and platelets; hemoglobin 45 per cent. Took pepto-mangan, but had to go home in two weeks, continuing the treatment. Four weeks later he wrote: "Am feeling better than for years; have gained flesh steadily since I left." (I am sorry not to have been able to examine his blood again.)

I have given pepto-mangan Gude to many other patients with benefit, but have no record of blood examinations. Among these was one case of chronic gastritis with hypochlorhydria complicating malaria; one of hysteria (male, æt. 19), three cases convalescent from opium treatment, two of senile debility, two of chronic nephritis, and one of tuberculosis.

NOTE.—Case three relapsed after returning home. Six months later she returned, and blood examination left no doubt of her having progressive pernicious anemia. Bone marrow, arsenic, and mercury had no effect. Pepto-mangan Gude was used again with slight temporary subjective improvement.—WM. KRAUSS, M.D., *Memphis Medical Monthly*.

---

### THE REMOVAL OF CERUMEN FROM THE EXTERNAL AUDITORY CANAL.

In a practical note upon this subject, we are told by Laurent that under no circumstances should cerumen be removed from the ear by the use of metal instruments, such as tweezers, stylets, or similar instruments which may do damage, unless the physician be an experienced otologist and familiar with the technique of removing foreign bodies from the ear; for, by the use of these instruments, aside from the ordinary damages which may be done by bruising, it is possible to produce furunculosis of the external auditory canal through infection of the glands, and there is also danger of injuring the tympanic membrane, or of causing hemorrhage from the canal, or deafness and vertigo. By far the safest and most efficient plan for the removal of wax under these circumstances is the use, by means of an absolutely sterile syringe, of water which has been sterilized by boiling and which is as hot as the patient can bear. If desired, a very small portion of carbolic acid may be added for its antiseptic influence. After the water is sucked up into the syringe, care should be taken that the point of the instrument is elevated and that all air is driven out of it, as the injection of bubbles of air into the external auditory canal produces a very disagreeable and painful sensation in the ear. A small pitcher or vessel, usually used for receiving liquid when the ear is being irrigated, is held tightly against the skin beneath the ear, then the water is injected with gradually increasing force until the mass is loosened and readily removed. Great care must be exercised that the first drops in particular are not driven into the ear with too much force, for if force is used vertigo and pain will result. In other words no forcible methods should be used under any circumstances for the removal of such accumulations as we are speaking of. Five or six syringefuls of water are usually sufficient. In other instances irrigation by means of a fountain syringe held eighteen inches or two feet above the ear of the patient is more efficient than the piston syringe. If the mass in the ear is so hardened that the injection of water will not soften it, it is well to prescribe as follows:

| | | |
|---|---|---|
| ℞ | Bicarbonate of sodium | gr. xv. |
| | Glycerine | ʒ j. |
| | Water | ʒ j. |

Three times a day the patient drops into the ear by means of a spoon, or with a medicine-dropper, five or ten drops of this solution, which has previously been warmed, and after it has remained there for a few minutes a pledget of cotton should be placed in the external auditory canal in order to preserve the moisture. In the course of a day or two the cerumen will be so softened by this solution that it will be easily removed. If it is perfectly free in the auditory canal it can, of course, be removed by a scoop or tweezers, but these are never to be employed unless the concretion is already loosened. After the removal of the mass the ear should be thoroughly dried by means of absorbent cotton, and a small pledget of absorbent cotton be placed in the external auditory canal to protect the part from cold. If these measures are used, serious results never occur, and the deafness which has been produced by the obstruction in the canal speedily disappears.—*Journal des Practiciens.—Pacific Record.*

### RETROVERSION OF THE UTERUS.

At the American Gynecological Society in Washington in May, Dr. Lapthorn Smith, of Montreal, gave the results of one hundred and forty-seven operations performed by him for retroversion of the uterus. His paper was based upon ninety-four ventrofixations and fifty-three Alexander's operations. He held that ventrofixation was the only operation that should be entertained in cases of retroversion with adhesions; but it should not be done when the uterus was movable and when there was no disease of the appendages requiring abdominal section, in which cases Alexander's operation had given excellent results. There should be no death rate to either operation, neither should there ever be hernia, either ventral or inguinal, if the following directions were followed. The two operations were equally easy, although a few years ago the author was opposed to Alexander's operation on account of its difficulty. Now he could invariably find the ligaments, and generally in from half a minute to a minute and a half. He warned his hearers not to do Alexander's operation if there were any adhesions, even if they were loose enough to permit the uterus to be lifted up; because they would be put upon the stretch and would drag so much upon the ligaments as to finally pull them out of their anchorage. In laying down the technique of Alexander's operation he placed great stress upon the importance of putting aside all cutting instruments as soon as the skin, superficial and deep fascia had been cut through. Instead of laying open the inguinal canal as advocated by some writers, he advised his hearers not to cut a single fiber of the intercolumnar fascia which was the principal support of the pillars. Moreover, he said, the slightest nick of the fascis of the internal oblique would lead to a false passage and failure to find the ligament. If no cutting instruments were used, but only a Peans forceps to draw out the ligament there would be no difficulty in finding it, because there was nothing else in the canal but the ligament. In fact, with the eyes bandaged it could be found and drawn out, simply by introducing the closed forceps and then opening them when the round ligament will fall into them and can be drawn out. He advocated the use of fine silk-worm gut, which could be thorougly sterilized and left in permanently. Occasionally he had been obliged to remove a buried stitch. In case any fibers of the intercolumnar or internal oblique should be accidently cut, great care should be exercised in sewing them up to avoid hernia. He had only had one relapse after ventrofixation and one after Alexander, which were both subsequently repaired. Several of the cases of ventrofixation had since become pregnant and had had normal confinements. Also several cases of Alexander had had children. Many of the patients had been bed-ridden invalids for years before and were now enjoying excellent health. Both operations, each in its proper sphere, had given the greatest possible satisfaction.

---

### EARLY SIGNS OF LOCOMOTOR ATAXIA.

According to Professor Fournier, the first symptoms of locomotor ataxia may be classed as follows: (1) Sign of Westphal; (2) sign of Romberg; (3) the "stairs" sign; (4) crossing of the legs; (5) walking at the word of command; (6) standing on one leg.

1. Westphal's sign is well known. It consists in the abolition of the patellar tendon reflex, and is present in two-thirds of the cases.

2. Romberg's sign can be thus appreciated: The eye is an indirect regulator of motion. It helps to correct deviations in walking and maintains the equilibrium. When a patient is suspected of incipient ataxia, it will often suffice to make him close his eyes when in the erect position to verify the diagonis. In a few instances his body will oscillate, and if the malady is somewhat advanced he will be in danger of falling.

3. The "stairs" symptom. One of the first and most constant symptoms of incipient locomotor ataxia is the difficulty with which the patient will descend stairs. If questioned closely on the subject, he will say that at the very outset of his malady he was always afraid of falling when coming down stairs.

4. The manner in which a patient crosses his legs is often significant. In the normal state, a man, when performing that act, lifts one leg simply to the height necessary to pass it over the other, whereas in the affection under consideration he lifts it much higher than necessary, describing a large segment of a circle.

5. Walking at the word of command. The patient seated, is told to get up and walk instantly. After rising he will hesitate, as if he wanted to find his equilibrium before starting off. If while in motion he is told to stop short, his body, obeying the impulsion, inclines forward as if about to salute, or, on the contrary, jerks himself backward in order to resist the impulsion forward.

6. The patient is asked to stand on one leg, at first with his eyes open, afterward closed. Although man is not made for this position, yet he can balance himself pretty firmly for a little while. The ataxic will experience a great deal of difficulty, and will instinctively call to his aid his other foot, so as not to fall. If his eyes are closed he will not be able to stand one instant, and if not held would fall heavily to the ground.

Such are the symptoms of incipient locomotor ataxia. They will not be all present frequently, but they should be all sought for in order to avoid an error which might have grave consequences.—*The Practitioner.*

---

### PUERPERAL ECLAMPSIA; ITS ETIOLOGY AND TREATMENT.

Though the pathogenesis of eclampsia is still unsettled, we are certain that it is a condition *sui generis,* pertaining only to the puerperal state, and that to describe, as formerly, three varieties—hysterical, epileptic and apoplectic—is erroneous as to pathology and causation as well as misleading in treatment.

The kidney plays an important office in the economy of the eclamptic. If it fails to eliminate toxins, symptoms are promptly presented in the pregnant woman. Renal insufficiency is a usual accompaniment of the eclamptic state. Overproduction of toxins and under-elimination by the kidney is a short route to an eclamptic seizure. However, many women with albuminuria escape eclampsia, and many eclamptics fail to exhibit albuminous urine.

The microbic theory of eclampsia has not yet been demonstrated. The toxemic theory, in the present state of our knowledge, furnishes the best working hypothesis for prevention or cure.

Treatment should be classified into a (*a*) preventive, and (*b*) curative. The preventive treatment should be subdivided into medicinal and hygienic; and the curative into medicinal and obstetric. A qualitative and quantitative analysis of the urine must be made at the onset. If there is defective elimination something must be done speedily to correct a faulty relationship between nutrition and excretion. One of the surest ways to control progressive toxemia is to place the woman upon an exclusive milk diet. This will also serve to flush the kidneys and thus favor elimination. Distilled water is one of the best diuretics; it increases activity and supplies material—two important elements. In the pre-eclamptic state, when there is a full pulse with tendency to cyanosis, one good full bleeding may be permissible, but its repetition should be regarded with suspicion. If there is high arterial tension—vasomotor spasm—glonoin in full doses is valuable.

When eclampsia is fully established the first indication is to control the convulsions. Full chloroform anesthesia may serve a good purpose. If the convulsions are not promptly controlled, the uterus must be speedily emptied. This constitutes the most important method of dealing with eclampsia. Two lives are at stake, and by addressing ourselves assiduously to speedy delivery of the fetus we contribute in the largest manner to the conservation of both.

Rapid dilation, first with steel dilators, if need be, then with manual stretching of the os and cervix, followed by the forceps, is the nearest approach to idealism. Only rarely can the deep incision of Duhrssen be required. Cæsarean section should be reserved for extreme complications as deformed pelvis, or to preserve the fetus when the mother's condition is hopeless. Veratrum viride is dangerous, uncertain and deceptive in action.

In eclampsia of pregnancy, *i. e.*, prior to term, the aseptic bougie, introduced to the fundus and coiled within the vagina, may be employed to induce labor. Finally, to promote the elimination of toxic material, diuresis, catharsis, and diaphoresis should not be forgotten; neither should the hot-air bath, nor the hot pack be overlooked.—Dr. W. W. Potter, *Albany Medical Annals*.

THE JANET ABORTIVE TREATMENT FOR GONORRHEA.

In a second communication on this subject, published in the *Centralblatt für innere Medicin* for October 10th, Dr. Berthold Goldberg, of Cologne, expresses his conviction that the early treatment of gonorrhea by means of frequent injections of a solution of potassium permanganate is a trustworthy means of aborting the disease. He gives a table of fourteen cases, in all but one of which he succeeded in curing the affection within so short a time as to justify the use of the term abortion.

Six of the patients were suffering from a first attack of gonorrhea, five were affected with a second attack, two had their third attack, and one was in his fourth. The treatment was begun in two days after the infection (twelve hours after the first symptom) in one case, in three days after the infection (twelve hours after the first symptom) in two cases, in six days after the infection (one day after the first symptom) in two cases, in seven days after the infection (one day after the first symptom in one case and an unnoted length of time in the other) in two cases, in eight days after the infection (one day after the first symptom in two cases and six days in one case) in three cases, in nine days after the infection (two days after the first symptom) in one case, and ten days after the infection (three and four days respectively after the first symptom) in two cases, and in twelve days after the infection (seven days after the first symptom) in one case.

Five injections sufficed to effect a cure in one case, six in three cases, seven in one case, eight in one case, nine in one case, eleven in one case, twelve in one case, thirteen in two cases, fourteen in one case, and fifteen in one case, while about thirty injections were employed in the case which, although finally cured, in about a hundred days, was not aborted. In that case the patient went from the seventh to the tenth day without treatment. As a complication, he had follicular prostatitis. The only complication in any of the other cases was bacteriuria in one case, which did not interfere with a prompt cure. The deep urethra was involved and had to be irrigated in six of the cases. The gonococci disappeared in one day in two cases, in three days in two cases, in five days in one case, in six days in one case, in ten days in one case, in eleven days in two cases, in twelve days in one case, in fourteen days in one case, in about fourteen days in one case, and in about a hundred days in one case—the one that was not aborted. In one case this detail was not recorded, but in that case all traces of the disease had disappeared in four days. In almost all the cases the urine ceased to show any signs of a discharge coincidently with the disappearance of the gonococci, but in three of the cases not until after a period of from three to six days; the urine was examined at intervals of from two to a hundred days after the subsidence of the disease. All the patients were allowed to go about as usual during the treatment.

When it was practicable, Dr. Goldberg gave the injections himself twice a day, but several of the patients were unable to present themselves so often, and consequently used the syringe themselves for a portion of the time, employing a solution of the strength of from 1 to 2,000 to 1 to 4,000 and injecting only the anterior urethra. Moreover, in several of the cases the treatment by the author himself had to be intermitted for as much as three days. All things considered, this seems to be a remarkably good showing for the Janet treatment.—*N. Y. Med. Journal.*

---

### HOW FAR CAN THE SURGEON GO?

In a recent case before the London courts, the question of how far a surgeon shall consider himself bound by the limitations set by the patient came up for determination.

Dr. Charles J. Cullingworth, an eminent London surgeon, performed the operation of double ovariotomy upon a patient at St. Thomas's Hospital two years ago. The plaintiff claimed that she distinctly forbade the surgeon to go beyond the single operation. She had been compelled in consequence to break off her marriage engagement. The defense claimed that she had given tacit consent to whatever operation might prove necessary. The jury rendered a verdict for the defendant.

In the course of the trial the defendant and Sir T. Spencer Wells, Lawson Tait, and other leaders of the profession, affirmed that they would refuse to perform any operation under such a restriction as the plaintiff declared she had imposed.—*Am. Med.-Surg. Bulletin.*

---

### THREE OBSTETRICAL WARNINGS.

There are three warnings which obstetricians should have constantly in mind, which are almost uniformly neglected:

First. Warn a woman not to neglect any kind of hemorrhage during pregnancy.

Second. Warn a woman during labor that she must keep her hands away from her vulva and vagina so long as she is confined to bed.

Third. Warn a nursing woman never to fall asleep with the infant at her breast.—DR. MABBOTT, *N. Y. Med. Journal.*

---

### TRACHOMA AMONG NEGROES.

Chibret of France has made an investigation with regard to the immunity of various races to trachoma, and among other conclusions he declares a relative immunity of the negroes of North America. This will certainly accord with the experience of most opthalmologists of this country, for, while we occasionally observe trachoma in the black race, it is exceedingly infrequent, and I believe that a more extended inquiry would establish a relative immunity to many other diseases of the eye.—*Courier-Rec. of Med.*

---

### ACUTE FOLLICULAR TONSILLITIS.

In the treatment of this condition Dr. E. Fletcher Ingals, of Chicago, recommends the application to the inflamed tonsil of a 50 per cent. solution of guaiacol in oil of sweet almonds. Internally:

℞ Potass. bromid.................................................gr. 80.

Sod. salicyl.................................................ʒ i.

Tr. opii deodoriz.................................................ʒ i.

Cascara cordial ad.................................................℥ i.

M. Sig. Teaspoonful every four hours in water.

—*Chicago Clinical Review.*

